# High fluency can improve recognition sensitivity based on learned metacognitive expectations

**DOI:** 10.3389/fpsyg.2022.958511

**Published:** 2022-09-20

**Authors:** Sarah Esser, Clarissa Lustig, Hilde Haider

**Affiliations:** Department of Cognitive Psychology 1, University of Cologne, Cologne, Germany

**Keywords:** fluency, metacognition, recognition, implicit learning, sensitivity, bias

## Abstract

Fluency of processing has shown to influence recognition judgments. Fluency most commonly induces a liberal response bias to judge fluently processed information as well-known because knowledge of a high correlation between the frequency of encounters, memory strength, and thus fluency of processing has been acquired in the past. In this study, we aimed to show that high fluency can increase recognition judgment sensitivity as well if the participants had encountered fluent and non-fluent processing during training. Thirty-three participants have been trained with a 12-element sequence in a serial reaction time task. During training, the response stimulus interval alternated block-wise between constant (fluent) and variable (non-fluent). Participants showed a higher capability of discriminating between old and new test sequences under fluent than under non-fluent test conditions. Furthermore, participants did not show any liberal or conservative bias after they have been trained with alternating fluency.

## Introduction

When we interact with the world, our actions, perceptions, and thoughts can be experienced with different fluency. When, for example, we try to recall the lyrics of our favorite song, this will subjectively feel easy. When we try to learn a new dance choreography the sequence of movements might first feel very influent and the feeling of fluency will increase with practice. Reading a long and new word can feel slow and influent. More generally, different internal or external cues can affect the perceived difficulty or ease of a task or a process and thus affect what is subjectively experienced as fluent. These cues can arise from motor, linguistic, encoding, and attention processes but are not limited to these (Oppenheimer, 2008). Thus, the quite general subjective experience of fluency can be a valuable metacognitive heuristic about our own current internal knowledge states or memory strength.

Research has repeatedly shown that high perceived fluency is associated with a bias towards rating something as well-known (Jacoby and Dallas, 1981; Koriat, 2000). It can lead to items being judged as more likeable (Reber et al., 1998) or to judging information as being of higher validity (Reber and Schwarz, 1999). Fluency can also be helpful to infer future internal knowledge states, when we can process something with high fluency, we can usually assume that it will also be easily remembered in the future (Undorf and Erdfelder, 2013). Accordingly, experienced fluency affects self-control strategies, such as the choice of which items to learn (Metcalfe and Finn, 2008) or how much time should be allocated to different items (Undorf and Ackerman, 2017). Hence, feelings of fluency are not exclusive to certain decision domains or the result of one specific underlying process, but are seen as the result of the monitoring of different cognitive processes (Oppenheimer, 2008). This article, however, will mainly focus on the feeling of fluency in recognition judgments.

Why do subjective fluency experiences affect such a broad range of decisions? The assumption of many researchers is that we do not have direct access to our internal signals such as memory strength, but that we have to infer these states from the observation of our behavior, which includes internal subjective experiences (Unkelbach and Greifeneder, 2013; Koriat, 2016). Fluency is seen as a subjectively accessible “proxy” for non-observable, inaccessible states like memory strength. In real life settings, there is a high correlation between frequency of past encounters and fluency; instances that appeared often, have a higher activation in memory, are quickly retrieved or processed and thus are experienced as fluent (Hasher et al., 1977; Hertwig et al., 2008). Hence, many studies could exploit this meta-knowledge about the correlation between feelings of fluency and memory strength and externally manipulate perceived fluency. For example, repeated presentation can make information processing feel increasingly fluent (Hasher et al., 1977). Likewise, the similarity of a stimulus and a mask (Mei et al., 2019), font size (Rhodes and Castel, 2008), or gradual clarification of a stimulus (Buchner et al., 1997) affect fluency and thus judgments of knowing and learning.

Yet, the fact that feelings of fluency are the result of various proximal cues like response speed or stimulus clarity has different implications for their validity and functionality. One implication is that they sometimes can be misleading, as shown by the many studies that induce a “fluency bias,” by which high fluency increases the likelihood of judging items as well-known (Jacoby and Dallas, 1981; Hertwig et al., 2008), even though they are, in fact, unknown. Likewise, participants can feel overconfident in being able to recall highly fluent items in the future (Finn and Tauber, 2015). A second implication is that there can be situations in which the individual knows that fluency is not a valid cue for memory strength and disregard it. For example, Kinder et al. (2003) proposed that whether perceived fluency is used as a cue for judging a test item is “known/old” or “unknown/new” is dependent on the induced judgment strategy. The authors found that perceptual fluency was irrelevant for knowledge judgments whenever participants had to use an *analytic strategy*. This meant that they had the rather difficult task to differentiate old from new test items even though some of the new items were structurally similar to the old items. In this case, participants might know that fluency is not a reliable cue because new items might also feel fluent. Similarly, Scott and Dienes (2010) found that fluency would only be used as a heuristic for knowledge judgments when there were not any other informative sources for a decision. Even if fluency *per se* is used as a heuristic cue, different contributing factor to the general feeling of fluency can be weighted differently depended on the situation. Previous research has shown that individuals can integrate multiple fluency cues for metacognitive judgments and strategically put more weight on certain fluency cues that they consider diagnostic for the specific situation (e.g., motor fluency; Undorf et al., 2018; Undorf and Bröder, 2020).

A third implication for the utility of feelings of fluency can be derived from the fact that high fluency does not necessarily imply high memory strength. The impact of fluency on decision-making is not only driven by momentary information, either from within the system (e.g., increased processing speed due to learning) or from outside of the system (e.g., font sizes, clarification speed, and judgment situation). Instead, fluency as a metacognitive experience itself is subjected to learning processes; currently experienced fluency is compared to expected fluency learned in similar past experiences. A comparison of expected and experienced fluency has been suggested previously by Whittlesea and Williams (2001a,b); discrepancy-attribution hypothesis and see also Benjamin et al. (1998). In their studies, test items that were perceived as fluent but were expected to be non-fluent, led to an increase of false-alarms or fluency bias. In these cases, participants seemed to assume that the best explanation for the unexpected perceived fluency was an encounter in the past.

Thus, individuals can use fluency experiences in a variety of ways, dependent on similar past experiences. High fluency can be a useful heuristic, if all past experiences granted fluent processing. This has, for example, been shown by Hansen and Wänke (2008) and Wänke and Hansen (2015) for a review. In their studies, high fluency only led to increased liking, positive attitude, or truth judgments when it was contrasted to low fluency during the test. Sudden non-fluent perceptions during the test are highly diagnostic of the situation being new and, likewise, any encounter that feels fluent can be interpreted as well-known, leading to a fluency bias. Unkelbach (2006) showed that the information gained from highly fluent processing can even be interpreted in a contrary way, depending on previous experiences. If past encounters were non-fluent, participants learned that a lack of fluency is a valid cue for having encountered something in the past. Thus, non-fluent test items would induce a bias for judging new test items as “old.” Similarly, Wilbert and Haider (2012) showed that typing-errors were most likely to be consciously detected when participants experienced typing as being less fluent than normal but also when they experienced it as being more fluent than normal. This further supports the idea that it is not high or low fluency *per se* that lends itself towards an interpretation, but that the difference in expected and experienced fluency matters. Any positive or negative deviation from the expectation can be interpreted in various ways, dependent on context and previous experiences. Hence, so far, research has shown that participants can learn that a specific fluency cue is diagnostic for memory strength. They can learn that either high or low fluency as indicative of high memory strength or that the same cue might be non-informative in a given situation.

What would be expected if an individual has had variable past experiences with fluency and learned that there can be situations where behavior or perception can feel fluent and that sometimes it can feel non-fluent? In these cases, the participant should be able to learn that neither high nor low fluency is diagnostic *per se* and should not be used as a mere heuristic. Nevertheless, we assume that this will not make fluency irrelevant for decisions and that the participant might very well still make use of it. We assume that high fluency can be useful to make more accurate decisions instead of simply biasing the judgment, if there is a training with varying degrees of fluency. Without such training, if the fluency manipulation is introduced during recognition tests, this manipulation is highly salient to the participant. It is likely that this unexpected perceived difference in fluency is attributed to varying degrees of memory strength. Under training with varying degrees of fluency; however, the cue that serves as the fluency manipulation (e.g., clarification speed or font size) *per se* should not be used for decisions because the participant was able to learn that this fluency variation is to be expected. Instead, the individual can put more weight on other, less salient behavioral experiences that influence the feeling of fluency, such as error rates, response speed, or correct predictions. Nevertheless, these behavioral indicators should mainly be informative under fluent test conditions. If fluent perceptual or motor performance is granted by an external fluency manipulation, the individual can experience finer differences in stimulus processing or response speed and thus, discrimination performance should be higher. If, however, external factors determine non-fluent, slow or irregular processing or responding, several other behavioral indicators for determining whether an item is old or new are also difficult to assess subjectively. In other words, high fluency is not only an often useful heuristic, it can also serve as an enabling circumstance to accurately interpret different behavioral cues such as response or perceptual speed, respectively predictions.

The current study will investigate how with fluency can aid recognition judgment accuracy, if the participants had experiences with varying degrees of fluency during training. To our knowledge, no other study so far has investigated this issue. Our hypothesis is that if participants are trained with fluent and non-fluent encounters of a repeating sequence, they will show better discrimination performance under fluent than under non-fluent test situations.

From a methodological point, this requires some contemplation about suitable paradigms. Two paradigms are commonly associated with the investigation of the influence of fluency on recognition decisions. One is learning of a list of paired associates, which allows for a broad range of manipulations of the study material (modality, semantics, learning effort and strategies, difficulty, etc.). In these paradigms, participants are explicitly asked to learn the associative pairs. These designs are, for example, particularly interesting when the aim is to investigate the influence of fluency on judgments of learning. A second frequently encountered paradigm involves implicit sequence learning. While there are different variations of this paradigm, such as the *Artificial Grammar Learning Task* (Reber, 1967) or the here employed serial reaction time task (SRTT; Nissen and Bullemer, 1987), they all share one major commonality: Participants are trained with stimuli and/or responses that follow a certain sequence. Participants are never informed about the sequence; they are simply instructed to attend or respond to the stimuli on the screen. In most cases, participants do not realize that the task contained a repeating sequence and are furthermore unable the recall the learned sequence. Nevertheless, implicit learning can be verified by showing slowed response times to new and unknown sequences or by better than chance recognition judgments (see Abrahamse et al., 2010, for review on implicit sequence learning). This design is often involved in fluency research for different reasons. For example, to investigate how intuitive judgments without any explicit knowledge base are influenced by fluency (Topolinski and Strack, 2009), to exploit fluency effects in order to infer what has been learned in an implicit learning situation (Ling et al., 2016), and, probably most commonly, to investigate two-process theories of learning and memory, where for example implicit memory processes are thought to be influenced by fluency, but not explicit processes (Buchner et al., 1997; Kelley and Jacoby, 2000; Kinder et al., 2003).

Here, we implemented an implicit learning paradigm because we found it to be the most practical approach for our goal to train participants with varying degrees of fluency. We aimed for a design in which the extent of internal signal strength due to learning is kept constant across all test situations so that varying memory strength should not be responsible for different recognition performance. Training participants with associative word pairs under different fluency contexts might lead to differences in the underlying knowledge base (e.g., non-fluent pairs might be learned to a lesser extent). However, if participants are trained with the same sequence repeatedly under varying fluency, it can be assumed that one underlying memory signal is developed under both fluency contexts. Moreover, this paradigm conveniently can remove or reduce idiosyncratic variances in associative pair learning that can affect metacognitive strategies (Undorf et al., 2022). Hence, with an implicit learning paradigm we aim to create a situation where the same memory signal can be associated with different degrees of fluency and repeatedly test how fluency manipulations during the test affect recognition judgments. A further benefit of this paradigm is that it can provide an opportunity to see whether fluency manipulations during training or test affect recognition decisions differently (Kinder et al., 2003).

## Materials and methods

### Training task

The implicit learning paradigm that was implemented is the SRTT. Participants were presented 6 white, horizontally arranged squares in the middle of the screen. These six squares were spatially mapped to six response keys on a keyboard, which were “z,” “x,” “c,” “b,” “n,” and “m” on a QWERTY layout. Accordingly, the leftmost square was mapped to the “z” key and so on. On each trial, one of the squares was filled with a target stimulus. Once the target stimulus appeared, it was the participants’ task to press the corresponding key as fast as possible, while also trying to avoid errors. Unbeknownst to the participants the target locations and thus also the motor response locations followed a 12-element second-order response-and stimulus-location sequence. That sequence was the same for all participants: b-c-m-n-x-z-m-b-x-c-n-z. The constraints for the construction of this sequence were that all six positions had to be used, before they could be used again in the second half; no transition was allowed to be repeated and it was not allowed that three adjacent keys were to be pressed. Participants were trained with 80 repetitions of this sequence. The resulting 960 trials were arranged in 10 training blocks á 96 trials.

Fluency was manipulated by varying the timing between the response and the appearance of the next target stimuli response stimulus interval (RSI). During half of the training blocks, the RSI was kept constant (380 ms) in order to create a fluent experience. Non-fluent processing was created by randomly varying the RSI (100, 190, 380, 660, and 750 ms). No two RSIs were allowed to be immediately repeated and all RSIs were to be used equally often. Fluent and non-fluent blocks alternated and it was counter-balanced whether participants started with fluent or non-fluent blocks. Manipulations of the RSI have previously successfully been used by Buchner et al. (1997) in an implicit learning situation to induce differences in feelings of fluency. Additionally, we ran a pilot study in our own lab with the material we used, in order to assess whether participants experienced fluency differences. In this pilot, participants judged the fluent trials as more likeable, preferred training with them, and, in subsequent interview, described them as feeling fluent. Several studies have demonstrated this association between perceived fluency and positive, affective ratings (Winkielman and Cacioppo, 2001).

**Figure 1:**
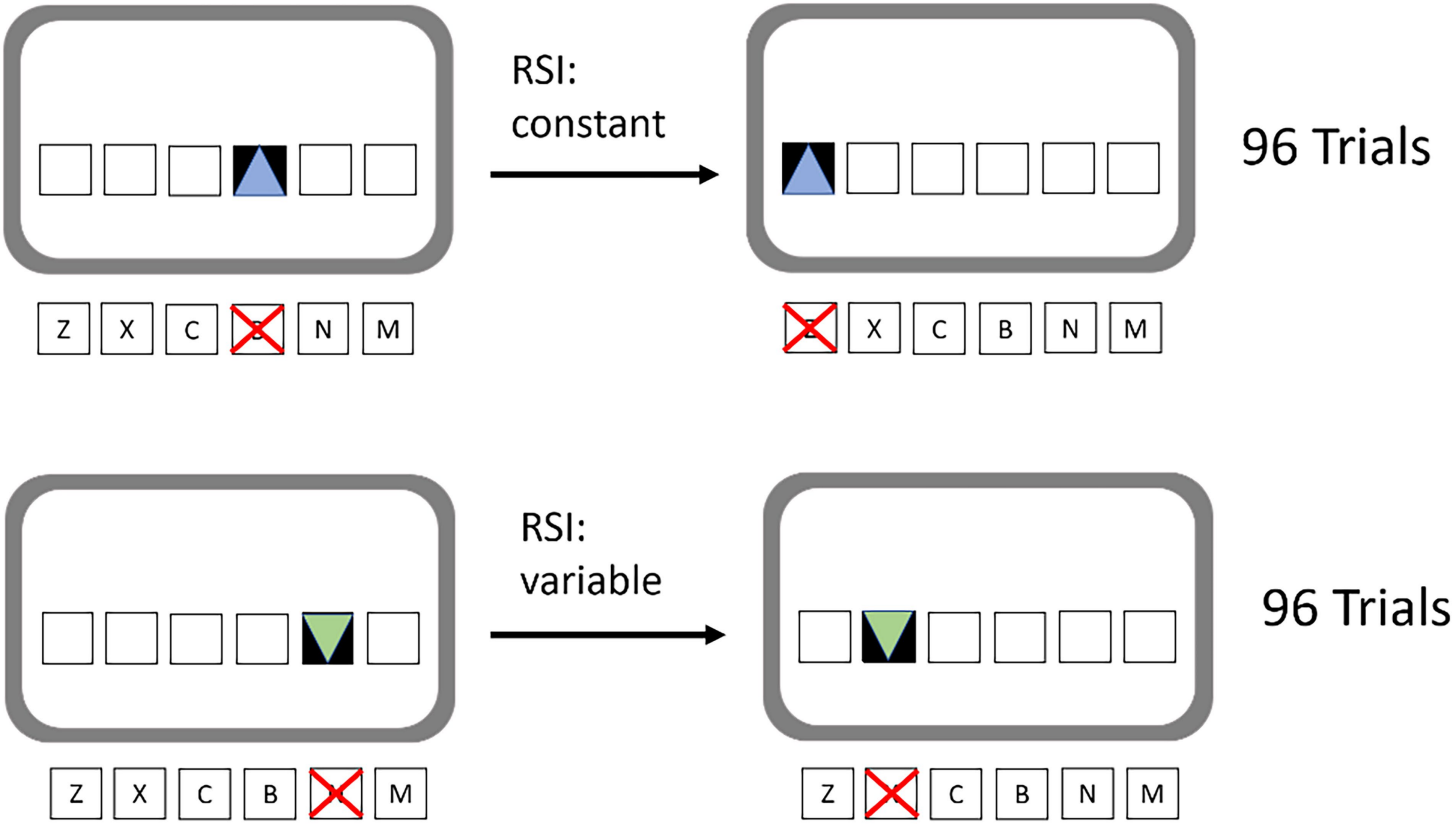
Schematic depiction of the training task. Colored target stimuli (triangles) were contingent with high (top) or low (bottom) fluency.

Additionally, fluent and non-fluent blocks were given a discriminatory color cue. The fluent or the non-fluent blocks were always associated with either blue or green triangles as target stimuli. It was counter-balanced between participants which color was associated with which fluency. This was done to provide the participants a context cue that can trigger fluency expectations in the later test phase. This way participants would be able to predict the expected fluency right at the start of each test block and compare the experienced fluency to this expectancy. The training task is presented schematically in Figure 1.

### Test task

In the test task, participants were informed that there was a repeating sequence and that in the following they would be presented short blocks that either contained the training sequence or a new one and that it would be their task to decide for each presented sequence whether it was “old” or “new.”

In total, the participants received 16 mini-blocks of 24 trials each. Half of these mini-blocks contained the original training sequence, starting with a random entry-point. The other half contained 12 structurally comparable new sequences. All participants received the same new test sequences but in different orders. Each mini-block either contained a new or an old sequence, presented either fluently or non-fluently. The order of all four types of mini-blocks was randomized and all four types had to be used before they could appear again.

At the beginning of each mini-block, participants were shown the stimulus-type of the following trials. These were the familiar blue or green triangles from the training task. This way, participants could anticipate the following fluency of the next mini-block. Before each mini-block, we also asked them to give a prospective judgment how sure they felt that they would be able to discriminate between a new and an old sequence, given the presented stimulus. They could rate their prospective confidence using a scale from 1 to 4 (1 = “certainly wrong,” 2 = “probably wrong,” 3 = “probably correct,” and 4 = “certainly correct”). This way, we could see whether participants learned to predict the fluency of the following trials and use this prediction to assess their performance capabilities. After the 24 trials of each mini-block, participants were first asked whether the sequence they were presented was “old” or “new.” They were additionally asked how sure they were about their answer. They could answer that question by using a scale ranging from 1 to 4 (“1 = certainly wrong,” “2 = probably wrong,” “3 = probably correct,” and “4 = certainly correct”). By asking this question we could get an impression about the influence of fluency on perceived judgment confidence.

### Sample

We collected our data online *via* the Prolific (2021) platform. Participation was compensated with a payment of 5 £. We collected the data of 33 participants (Mean age = 32.8, *SD* = 11.9). Sample size was based on a GPower 3.1 analysis for our design with a medium effect size, *α* = 0.05 and 1 − *β* = 0.95. All participants were 18 years or older and gave informed consent before the start of the experiment.

We excluded participants with more than 15% erroneous trials during training or test. This led to the exclusion of six participants. Also, we excluded one participant because they always gave the same recognition judgment during the test task. Due to these selection criteria 26 participants were included in the analysis.

## Results

### Data analyses

First, we analyzed the reaction time data of both the training and the test task. These analyses, even though not the primary focus, can provide interesting insight into the behavioral effects of our fluency manipulation during training and test. Moreover, reaction time differences between old and new sequences during the test can be seen as an indicator of sequence learning. For the RT analysis, we removed all erroneous trials. We also removed any trial that followed an error due to post-error slowing and the circumstance that any keypress after an erroneous one is also incorrect in terms of its associative link to the previous trial.

Our main interest lies in the subsequent signal-detection-based analyses of the “old”/“new” recognition judgments during the test task and how fluency manipulations during the test task influenced these decisions. According to our hypothesis under fluent test conditions, accuracy (sensitivity *d*′) should be improved, while the response bias (*c*) should not depend on fluency.

Additionally, we will investigate, exploratively, whether participants have some subjective insight into the beneficial effects of fluent task processing by analyzing their confidence judgments before and after the test sequences were presented. Furthermore, analyzing the confidence judgments after the recognition decision can provide an opportunity to exploratively discriminate between participants with rather implicit or rather explicit sequence knowledge. Thus, we will also analyze whether fluency manipulations during the test phase affect sensitivity and bias differentially for participants with implicit and explicit sequence knowledge.

### Training task: Reaction time data

We pooled the data of each two of our 10 training blocks that contained a fluent and a non-fluent block. This resulted in an ANOVA with Block (1–5) × Trial Type (fluent vs. non-Fluent) as repeated within-subject factors.

**Figure 2:**
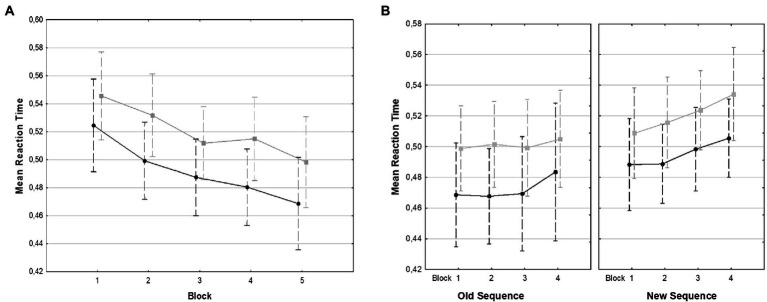
Mean reaction times for all training **(A)** and test **(B)**. Blocks: black lines (circles) show RT for fluent trials, gray lines (squares) show RT for non-fluent trials. Bars denote 95% confidence Intervals.

We found a main effect for Block [*F*(4,100) = 19.15, *p* < 0.001, $\eta_{p}^{2}$ = 0.43]. A linear contrast showed that RT decreased with each block [*F*(1,25) = 28.74, *p* < 0.001]. We also found a main effect or Trial Type with fluent material being associated with faster RT than non-fluent trials [*F*(1,25) = 49.20, *p* < 0.001, $\eta_{p}^{2}$ = 0.66, mean RT for fluent trials: *M* = 492.00 ms, *SD* = 69.12, mean RT for non-fluent trials: *M* = 520.45 ms, *SD* = 69.12]. The Block × Trial Type interaction was not significant (*F* = 1). Reaction time data for training are shown in Figure 2A.

### Test task: Reaction time data

In the test task, new and old sequences were presented in either the fluent or the non-fluent material. We analyzed RT in a repeated-measures ANOVA with Fluency (fluent vs. non-fluent trials), Sequence Type (old vs. new), and Block (1–4; the 16 test blocks were pooled in groups of 4) as within-subject factors. There was a significant main effect for Block [*F*(3,75) = 4.16, *p* = 0.008, $\eta_{p}^{2}$ = 0.14]. A linear contrast showed that RT increased with each test block [*F*(1,25) = 9.95, *p* = 0.004]. Fluent trials were associated significantly shorter RT than non-fluent trials [*F*(1, 25) = 116.11, *p* < 0.001, $\eta_{p}^{2}$ = 0.82, mean RT for fluent trials *M* = 483.75 ms, *SD* = 69.88, mean RT for non-fluent trials *M* = 510.86 ms, *SD* = 66.59]. Old sequences were associated with shorter RT than new sequences [*F*(1,25) = 14.28, *p* < 0.001, $\eta_{p}^{2}$ = 0.36, mean RT for old sequences *M* = 486.71 ms, *SD* = 74.80, mean RT for new sequences *M* = 507.90 ms, *SD* = 63.62]. No interaction was significant (all *F*s < 1). Reaction time data for the test are shown in Figure 2B.

### Test task: Recognition judgments

Our main aim was to test whether fluent processing conditions lead to a better ability to discriminate old from new sequences. Thus, we calculated two *t*-tests for dependent samples, for which Fluency (fluent vs. non-fluent trials) was the independent variable. The dependent variables were discriminatory performance, measured *via* sensitivity (*d*′) and response bias (*c*). Larger sensitivity scores indicate better abilities to discriminate between old and new sequences. A positive bias indicates a tendency to judge sequences as “new,” a negative bias a tendency to judge sequences as “old.” Because cells with 100% or 0% responses pose a problem to calculating *d*′ and *c*, we replaced rates of 0 with 0.125, respectively rates of 1 were replaced with 0.875 (Stanislaw and Todorov, 1999). Thus, the highest possible *d*′ is 2.3 and *c* can vary between −1.15 and 1.15. Table 1 shows the amount of “old” and “new” responses towards old and new test sequences separately for non-fluent and fluent presentations.

**Table 1:** Numbers and percentages of hits, false alarms, misses, and correct rejections for fluent and non-fluent conditions.

	Non-fluent		Fluent	
	Response “old”	Response “new”	Response “old”	Response “new”
Old sequence	56 (60%)	48 (40%)	68 (71%)	36 (29%)
New sequence	38 (38%)	66 (62%)	28 (32%)	76 (68%)
Sensitivity and criterion	*d*′	*c*	*d*′	*c*
	0.42 (0.99)	−0.11 (0.46)	0.93 (0.89)	−0.08 (0.40)

Bottom row shows mean sensitivity *d*′ and response criterion c for fluent and non-fluent conditions; standard deviations are reported in brackets.

We found no influence of Fluency on the response bias *c* (*t* (25) < 1). With regard to sensitivity, fluent test sequences were associated with better discriminatory performance than non-fluent test sequences [*t* (25) = 2.41, *p* = 0.015, one-sided]. As these two analyses were the most relevant to our hypothesis, we also calculated Bayes factors. These confirmed substantial evidence for the H0 regarding the response bias (*BF*_01_ = 4.66) and substantial evidence in favor of the H1 with regard to sensitivity (*BF*_+0_ = 4.58).^1^ Table 1 shows sensitivity and response bias data for fluent and non-fluent trials.

### Test task: Confidence ratings

Prospective confidence ratings were analyzed in a repeated-measure ANOVA with Block (1–4) and Fluency Cue (fluent vs. non-fluent cues) as repeated within factors. This revealed a non-significant tendency for Fluency Cue [*F*(1,25) = 3.89, *p* = 0.059, $\eta_{p}^{2}$ = 0.14]. Participants appeared to give slightly higher prospective confidence ratings for fluent (*M* = 2.86, *SD* = 0.69) than for non-fluent cues (*M* = 2.79, *SD* = 0.71). The main effect for Block and the Block × Fluency Cue interaction were not significant (both *F*s < 1.2).

For the subjective confidence judgments after the participants made their recognition decision, an ANOVA with Fluency (fluent vs. non-fluent trials), Correctness (correct vs. wrong classification) and Sequence Type (old vs. new) as within-subject factors was calculated. There was a significant main effect for Correctness, with correct answers being associated with higher confidence ratings [*F*(1,408) = 6.85, *p* = 0.009, $\eta_{p}^{2}$ = 0.017]. The mean confidence rating for correct responses was *M* = 2.67 (*SD* = 0.89), and the mean for incorrect responses was *M* = 2.42 (*SD* = 0.89). No other main effect or interaction was significant (all *F*s < 1).

### Explorative analyses for implicit and explicit sequence knowledge

The higher confidence ratings for correct than for incorrect responses could be seen as a hint that some participants acquired at least partially explicit sequence knowledge. As argued by Dienes and Perner (2002) explicit knowledge can be conceptualized as knowing that one knows. Hence, explicit knowledge can be conceptualized a giving more “correct” judgments on correct classifications than “correct” judgments on incorrect classifications.

For the following analysis, we classified subjective “correct” ratings as giving a post-judgment confidence score of either “3” (“probably correct”) or “4” (“certainly correct”). We then calculated an “explicit knowledge score” for each participant. We calculated whether they gave more subjective “correct” ratings after correct classifications than after incorrect classifications (i.e., number of “correct” ratings after a correct classification divided by the total number of “correct” ratings). Any participant with a score > 0.5 was classified as having some explicit knowledge. This resulted in nine participants having scores of ≤0.5 and 17 participants having scores >0.5 (*M* = 0.62, *SD* = 0.22). Because these groups were small, of uneven size and a very liberal criterion has been used to classify someone as having explicit knowledge, all following results should be interpreted with high caution and only be seen as an outlook for follow-up studies. Two ANOVA have been calculated. In both analyses, Fluency (fluent vs. non-fluent trials) was a repeated-measures factor and Knowledge Status (implicit vs. explicit) was treated as a between-subject factor. The dependent variables were sensitivity *d*′ and response criterion *c*.

**Figure 3:**
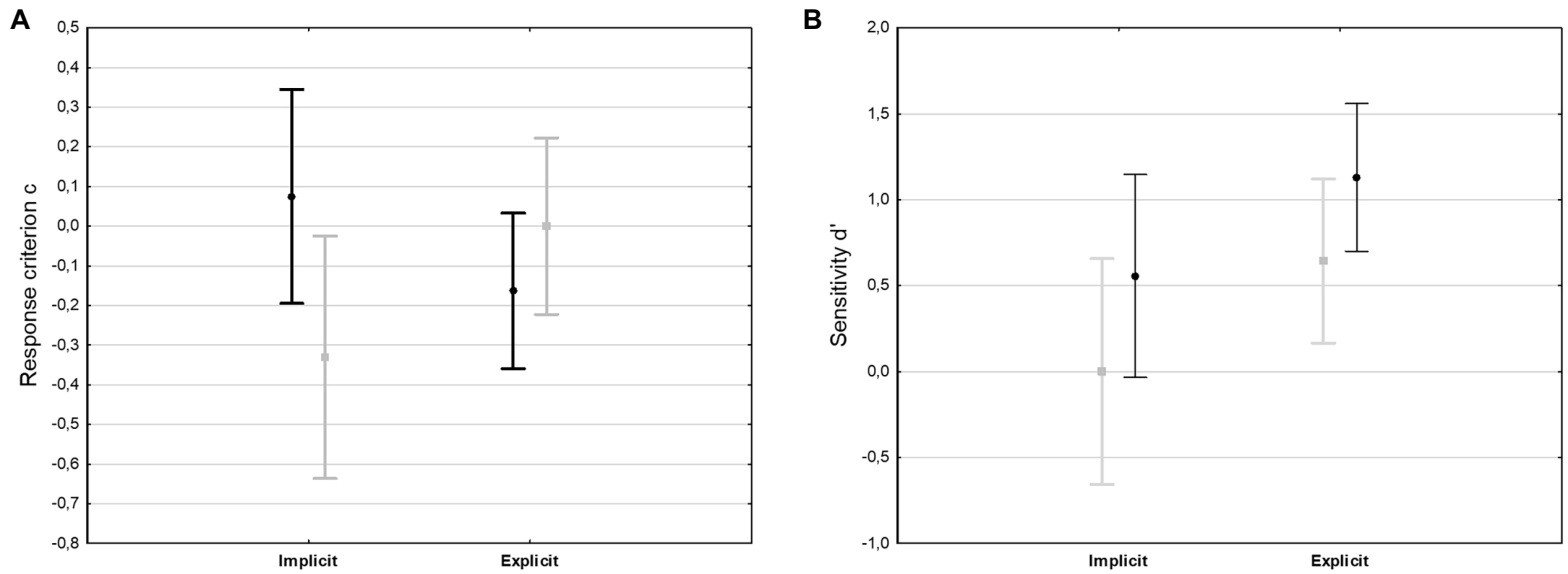
Differences in recognition response criterion c **(A)** and sensitivity *d*’ **(B)**. Between high (black, circles) and low (gray, squares) fluency sequences. Bars denote 95% confidence intervals.

For response criterion *c,* this analysis showed no main effect for either Fluency or Knowledge Status (both *F*s ≥ 1). There was significant interaction between Knowledge Status and Fluency [*F*(1,24) = 5.84, *p* = 0.024, $\eta_{p}^{2}$ = 0.02]. This interaction was the result of the implicit group showing a slight conservative bias under non-fluent conditions (*p* = 0.04). The explicit group did not show any difference for the different fluency conditions (*p* = 0.25). As can be seen in Figure 3A, no group showed a liberal response bias. This could be seen as a hint that participants with implicit sequence knowledge experience more uncertainty under non-fluent presentation and tend to reject non-fluent sequences.

For sensitivity *d*′, there was a significant main effect for Knowledge Status [*F*(1,24) = 4.18, *p* = 0.05, $\eta_{p}^{2}$ = 0.15] because participants with explicit knowledge had a better classification performance. Importantly, there also was a significant main effect for Fluency [*F*(1,24) = 5.28, *p* = 0.03, $\eta_{p}^{2}$ = 0.18]. Both groups with implicit and explicit knowledge showed improved discriminatory performance under fluent processing conditions. There was no interaction for Knowledge Status and Fluency (*F* < 1). Figure 3B gives an impression about the influence of both factors on sensitivity.

## Discussion and outlook

In summary, we found that participants were better at discriminating between old and new sequences when these were presented fluently. At the same time, fluency did not induce a liberal or conservative bias. Participants showed a non-significant tendency to put more predictive confidence in their performance on fluent trials and put significantly more confidence in correct judgments retrospectively. On a very cautious note, we found hints that this positive influence of high fluency after training with varying fluency is present for participants with implicit and with explicit sequence knowledge.

These results have different implications. Most importantly, this, to our knowledge, is the first study to show that fluency can lead to better sensitivity without inducing a response bias, if the participants made the prior experience that the task can vary in fluency. This finding extends previous theories about the validity and usefulness of fluency cues. Earlier research has already successfully shown that the use of fluency cues is more than a shallow heuristic by which high fluency is attributed to past encounters. It is an adaptively useable cue that gains its validity through comparison with past experiences in a given context (Whittlesea and Williams, 2001a,b; Unkelbach, 2006; Wänke and Hansen, 2015). In these prior studies, fluency showed its adaptive influence in an ecologically valid bias. Here, we demonstrated that fluency can additionally aid recognition sensitivity without inducing a bias.

We have argued that this positive influence on recognition performance is due to the higher weighting of less salient performance cues that are most noticeable under fluent task conditions. As other researchers (Undorf et al., 2018) have shown, participants seem to be able to strategically put more weight on different cues that influence fluency feelings. If highly salient fluency manipulations are first introduced during the test, these manipulations might have a strong weight in the general experience of fluency, which in these cases would drastically increase false alarm rates. The general idea is that different tasks could be associated with different and maybe even task-specific cues that influence feelings of fluency (Topolinski, 2013). In a motor task like the SRTT, manipulating the fluency of stimulus presentation and motor responses, like we did by manipulating the RSI, probably has a strong effect on fluency perception and thus recognition judgments. Without prior fluency training, introducing such a manipulation in the test probably has a very high weight for the participants’ feeling of fluency and induces a bias.

Training participants with the experience that the fluency of stimulus presentations and motor responses can vary, will lead to decreasing the weight of this otherwise important cue. Likewise, other more subtle cues can be regraded. However, processing these subtle cues might require certain boundary conditions, such as high fluency in stimuli and responses, granted by the task. Here, we found that old sequences were generally associated with shorter reaction times, which, for example, might be one important cue to tell apart new from old sequences. Yet, these small RT differences might remain unnoticed in the face of very non-fluent task conditions and are likewise more noticeable under fluent task conditions. Another cue within our implicit learning design could be correct motor predictions (Van den Bergh et al., 1990) that can be noticeable if the next target appeared in a certain, predictable time frame. If the time frame for the appearance of the target is unpredictable, too long or too short, as it is the case under non-fluent processing, these motor predictions probably remain subjectively unnoticed. In other task designs, like associative pair-learning other cues could be of higher salience when processing is fluent. For example, Topolinski (2013) argued that fluency perception of verbal stimuli is strongly influenced by oral motor-fluency and that in case of oral motor interference other cues such as visual fluency are disregarded. Our results suggest that if these participants were taught that oral motor fluency can vary, visual fluency might influence judgments, but only if oral motor fluency is high. Otherwise, low oral motor fluency could hinder the perception of the more subtle visual fluency.

There is also another interesting possibility on how training with varying degrees of fluency can aid recognition accuracy, which has not been investigated here, but could be the matter of future studies. If a variation in task fluency is first introduced during recognition tests, the participant assumes that there is a strong correlation between the task’s fluency and memory signal strength. They will base their recognition judgment on two distributions of experienced fluency signals. One signal distribution (assumed fluency of “old” items) and one noise distribution (assumed fluency of “new” items). Additionally, one decision criterion will be applied and whenever the experienced fluency is higher than the set criterion value, any item will be judged as “old”; whenever fluency is lower, it will be judged as “new.” In fact, when fluency is only manipulated during the test, unbeknownst to the participant, fluency and memory strength are only weakly correlated and high fluency can be associated with new and old items. In other words, the participant has wrong assumptions about the underlying signals and their interpretation. Introducing a fluency manipulation during the test leads to many false alarms (judging new items as old) because many new items are also perceived with high fluency and the participants’ interpretation is that this internal fluency signal is most likely when memory strength is high. Likewise, many misses (judging old items as new) are the consequence of old items that suddenly appear with low fluency.

However, through training with both, fluent and non-fluent encounters, the individual learns to apply two criteria (one for fluent and one for non-fluent encounters) to four different signals (fluent and non-fluent old) and noise (fluent and non-fluent new) distributions. The participant expects a certain, rather high threshold for fluent encounters that needs to be exceeded in order to classify an item as “old.” Many fluent encounters do not cross this threshold and are classified as “new.” This comprises a large proportion of actually new items. Thus, a proportion of high fluency experiences that previously would have led to a false alarm now are rejected correctly. For low fluency, only particularly low fluency is judged as “new” and the upper end of low-fluency could be judged as “old.” This avoids a significant number of misses for non-fluent but old items that now can be correctly classified as “old.” Taken together, training with fluent and non-fluent encounters helps the participant to learn that there are in fact different fluency signal distributions that can both be informative about the memory strength in different situations. Higher sensitivity is the result of less fluent but new items being classified as “old” and less non-fluent but old items being classified as “new.”

The current study also provides two explorative but possibly interesting side findings that can be interpreted tentatively. First, the participants showed signs of awareness about the beneficial effect of high fluency on recognition judgments. For prospective confidence judgments, fluent stimuli gave rise to higher confidence ratings, even though this effect did miss level of significance and should be interpreted with caution. The participants seemed to acknowledge that high fluency generally made their recognition decision more accurate, while at the same time they were not or not entirely conscious about the structural sequence knowledge that they had to judge. This could further strengthen the idea that metacognitive strategies can voluntarily and strategically be applied in intuitive decisions where the information that forms the basis for a decision itself is not conscious (Topolinski and Strack, 2009). Future studies could furthermore focus on the level of consciousness of the applied metacognitive strategies themselves (see e.g., Geurten et al., 2015).

At the same time, this knowledge about the benefits of fluent processing did not lead to a bias that increased post-judgment confidence. This underlines the missing liberal bias in recognition judgments. Neither did participants rate fluent sequences more frequently as old, nor did they express higher confidence after decisions made about fluent sequences. Their retrospective confidence only correlated with the correctness of their decision, signaling that the participants had a rather accurate assessment of their own knowledge status, both under fluent and under non-fluent conditions. This aligns our argument about the influence of fluency on recognition judgments with research on the influence of fluency on confidence judgments. Fluency can bias confidence judgments (Kelley and Lindsay, 1993). Yet, studies have shown that various cues can be used for confidence judgments and these cues can or cannot align with the cues used for recognition judgments (Koriat, 1997). High correlation between confidence and accuracy is achieved, if recognition and confidence judgments rely on the same cues (Busey et al., 2000; Reinitz et al., 2011). Thus, if, in our study, participants relied less on the task’s fluency *per se*, it is plausible that they also refrained from using it as a cue for their subjective confidence ratings.

A second interesting explorative finding was that a rough bisection of the participants into implicit and explicit sequence learners showed a highly comparable beneficial effect of high fluency on sensitivity. This supports the idea that metacognitive knowledge is not automatically applied to implicit or unconscious knowledge in the form of a simple fluency bias. Instead, the application of metacognitive knowledge is dependent on past experiences with similar situations. Again, future studies can take a closer look at the ability to adaptively use metacognitive knowledge in the absence of consciously available knowledge.

Certainly, this single study should be interpreted with caveats and some methodological issues should be addressed. Our study does not provide any direct evidence for any of the two above named explanations for increased sensitivity. This study cannot assess whether fluency is indeed increased under fluent conditions by the higher weighting of other, more subtle metacognitive cues. It could also or additionally be decreased under non-fluent conditions by deteriorating the signal-to-noise ratio. This needs to be addressed in future studies. Furthermore, we can only speculate about what could constitute helpful cues under fluent task processing that inform a participant that their performance is based on knowledge acquired in the past. Future studies will have to take a closer look at the information that increased participants’ accuracy under fluent processing. It is also open whether these other behavioral cues are integrated to a general, unspecific feeling of fluency or contribute to other metacognitive performance judgments than can exist in parallel to the perception of the externally manipulated fluency. Our other suggestion that fluency increases sensitivity by setting two comparison standards needs to be tested in future studies. These studies should reveal that sensitivity is increased in comparison to training conditions where all encounters are either fluent or non-fluent.

In summary, we suggest that fluent test conditions can further increase sensitivity, because fluent processing provides more opportunities to evaluate other cues, like response speed or motor predictions. Future studies can take a closer look at the degree to which other cues are used under fluent and non-fluent processing, given that training and test items match in their fluency. Lastly, our findings also need to be replicated in other fluency paradigms and applied settings. We propose that future studies should investigate whether and how training with varying fluency levels helps individuals to calibrate their metacognitive standards for recognition and possibly other judgments (liking, truth, etc.). This should include applied settings like self-controlled learning (Reber and Greifeneder, 2017) or eyewitness testimonies (Chua et al., 2012). Both settings aim for improving judgment accuracy by calibrating metacognitive accuracy. In both settings, cues should be identified that have a high influence on feelings fluency within the respective specific situation, but often lead to a bias that can be detrimental to accuracy. If, in these cases, individuals are confronted with situations that help them experiencing that the fluency of these cues can vary within the same context, they might gain accuracy by putting more weight on less salient cues that have a higher predictive value in the specific setting.

## Data availability statement

The raw data supporting the conclusions of this article will be made available by the authors, without undue reservation.

## Ethics statement

The studies involving human participants were reviewed and approved by Ethics Committee of the University of Cologne. The patients/participants provided their written informed consent to participate in this study.

## Author contributions

SE worked on theoretical conceptualization, study design, and data analysis, and wrote the article. CL worked on theoretical conceptualization, study design, and data analysis. HH worked on theoretical conceptualization and study design and helped writing the article. All authors contributed to the article and approved the submitted version.

## Funding

This research was funded by the Deutsche forschungsgemeinschaft (DFG), grant number: HA 5447/12–1.

## Conflict of interest

The authors declare that the research was conducted in the absence of any commercial or financial relationships that could be construed as a potential conflict of interest.

## Publisher’s note

All claims expressed in this article are solely those of the authors and do not necessarily represent those of their affiliated organizations, or those of the publisher, the editors and the reviewers. Any product that may be evaluated in this article, or claim that may be made by its manufacturer, is not guaranteed or endorsed by the publisher.

## Footnotes

^1^For both *t*-tests the prior is described by a Cauchy distribution centered around zero, with a width parameter of 0.707. A Bayes factor of 1/10 to 1/3 can be interpreted as substantial evidence for the H0, a Bayes factor of 1 provides no evidence, a Bayes factor between 3 and 10 can be interpreted as substantial evidence for the H1, and a Bayes factor > 30 as very strong evidence for the H1 (Jeffreys, 1961).
